# Improving critical care nurses’ knowledge of central venous catheter management through education

**DOI:** 10.4102/hsag.v30i0.2966

**Published:** 2025-07-09

**Authors:** Tembisa Notshe, Wilma ten Ham-Baloyi, Sindiwe James

**Affiliations:** 1 Department of Nursing Science, Faculty of Health Sciences, Nelson Mandela University, Gqeberha, South Africa

**Keywords:** critical care unit, central venous catheter, critical care nurses, knowledge, educational intervention

## Abstract

**Background:**

Central venous catheters are essential in critical care, but mismanagement risks serious complications, making comprehensive nursing knowledge crucial for their proper use and for safeguarding patient safety.

**Aim:**

To describe the effect of an 8-week educational intervention on critical care nurses’ knowledge of central venous catheter management.

**Setting:**

Five critical care units in public hospitals in the Eastern Cape, South Africa.

**Methods:**

A quasi-experimental, two-group, pre-test-post-test design was used with a convenience sample of nurses working in selected critical care units (intervention *n* = 72; control *n* = 50). The intervention group received an 8-week educational intervention including face to face in-service training sessions, written material (brochures) and monitoring visits twice a month, while the control group did not receive this training.

**Results:**

Both groups showed significant knowledge improvements (*p* < 0.0005), with the intervention group improving more during catheter insertion (+33.7 vs. +23.8; *t* = –16.68 vs. –5.59) and the control group more after catheter insertion (+33.3 vs. +21.5; *t* = –7.66 vs. –12.43). The intervention group had significantly higher mean pre-test scores (pre-test: 97.13; post-test: 98.01) compared to the control group (pre-test: 82.91; post-test: 83.76).

**Conclusion:**

The educational intervention showed potential to improve critical care nurses’ knowledge of central venous catheter management but require further adaptation and testing. Orientation, mentoring and continued formal training through institutional support and leadership are recommended.

**Contribution:**

The use of an 8-week educational intervention can improve critical care nurses’ knowledge of management of central venous cathether management and may enhance practices, ultimately reducing venous catheter-related complications.

## Introduction

The use of central venous catheters in critical care units cannot be avoided, and it is estimated that between 50% and 80% of critically ill patients need central venous access at some point during their stay in the intensive care unit (Zhang et al. 2018). Central venous catheters are crucial in creating the necessary systemic access for the purposes of administering large quantities of resuscitative fluids, parenteral nutrition fluids, vasoconstrictors or veno-sclerotic drugs, potassium solution medications, chemotherapy drugs, corrosive medication that damages peripheral veins, monitoring of haemodynamic status, hemofiltration and blood transfusions (Zhang et al. 2018). The three main access sites for central venous catheters are the internal jugular, common femoral and subclavian veins (Shah & Berman 2023). The types of catheters include tunnelled central venous catheters, which provide long-term vein access; peripherally inserted central catheters (PICC), such as implantable ports and central lines and subcutaneous (implanted) ports, also known as portacaths. These are commonly used for chemotherapy, medication administration or blood sample collection (Johns Hopkins Medicine 2025).

However, central venous catheters carry the risk of various complications. These may occur during insertion – such as vascular (e.g. arterial puncture, haematoma), cardiac (e.g. arrhythmia, cardiac arrest) and pulmonary complications (e.g. pneumothorax, tracheal injury) – or may arise later as delayed complications, including device malfunction, venous thrombosis and infections such as central line-associated bloodstream infections (CLABSIs) (Kornbau et al. 2015). Although the overall complication rate is estimated at between 1% and 34% (Adrian et al. 2022), insertion site infections and venous thromboembolism are two common and severe complications related to central venous access (De Grooth, Hagel & Mimoz 2024; Wall, Moore & Thachil 2016). These two complications increase morbidity, often necessitating premature catheter removal and treatment discontinuation, which in turn contribute to longer hospital stays, higher resource utilisation and increased healthcare costs (Haddadin, Annamaraju & Regunath 2022). Nurses are responsible for maintaining and monitoring central venous catheters; recognising complications like infections, thrombosis and pneumothorax and ensuring site sterility. Their role is crucial in identifying both immediate and delayed complications, making them essential members of the interprofessional team in managing central venous catheters (Kolikof, Peterson & Baker 2025).

### Background

To prevent poor management of central venous catheters and subsequently complications, the implementation of and adherence to clinical practice guidelines by critical care nurses are crucial (Zhou et al. 2025). Effective implementation and adherence to clinical practice guidelines for central venous catheter management require a thorough understanding of the recommended practices, including clinical indications, insertion and removal techniques and post-insertion care (Kolikof et al. 2025; Pereira et al. 2022).

During the study period, the first author observed that critical care nurses in the study setting had a notable lack of knowledge when it came to recommended practices for managing central venous catheters. The absence of best practice guidelines for central venous catheter management, coupled with a lack of training, has likely contributed to the lack of compliance with recommended practices. This gap in knowledge may explain the suboptimal practices observed among nurses in the study context as also found in other studies (Bibiano Guillén et al. 2024; Yang et al. 2024; Zhou et al. 2025). These practices included infrequent lumen flushing, leading to clotting and treatment discontinuation, as well as failure to adhere to proper hand hygiene and skin cleaning with chlorhexidine during and after insertion. Such practices, observed by nurses in the study, increase the risk of insertion site infections (Zhang et al. 2018).

Educational interventions can be delivered to individuals or groups, either face to face or via telephone, across various settings such as communities, hospitals, homes, schools and organisations. These interventions may use verbal, written or audiovisual methods, – including printed materials, multimedia tools (e.g. videos, PowerPoint presentations), counselling sessions, practical demonstrations, lectures and role plays. Typically grounded in clinical practice guidelines, these educational interventions often incorporate diverse teaching strategies and resources such as classroom instruction, computer lab sessions, structured courses, projects and counselling (Ciciriello et al. 2013; Melender, Mattila & Haggman-Laitila 2015; Nkhoma, Seymour & Arthur 2015). Educational interventions have demonstrated effectiveness in enhancing critical care nurses’ knowledge regarding central venous catheter management, ultimately leading to improved practices (Inchingolo et al. 2019). For instance, research in Jordan and India demonstrated that structured education and similar interventions were beneficial in increasing nurses’ knowledge regarding the nursing management of central venous catheters (Deshmukh & Shinde 2014; Pushpakala & Ravinath 2014; Sharour et al. 2018).

The study was conducted to describe the effect of an 8-week educational intervention on critical care nurses’ knowledge of central venous catheter management.

## Research methods and design

### Design

This educational intervention study employed a quasi-experimental, two-group pre-test–post-test design, consistent with methodologies employed in prior research (Alayemi, Ten Ham-Baloyi & Jardien-Baboo 2024) and was conducted from July 2020 to December 2020.

### Setting

This study was conducted at five public urban hospitals, three tertiary and two regional hospitals, situated in one of the most socio-economically disadvantaged provinces in South Africa (Booysen et al. 2024). Across the five urban hospitals, the total critical care bed capacity is 68, supported by 122 nurses. The tertiary hospitals – larger and better resourced – provide specialised care as follows: Hospital 1 (24 beds, adult general and high care), Hospital 2 (6 beds, adult and paediatric coronary care) and Hospital 3 (16 beds, adult and paediatric general and high care). Regional hospitals, which are medium sized with relatively limited resources, include Hospital 4 (12 beds, paediatric and neonatal care) and Hospital 5 (10 beds, adult and paediatric general care).

### Population and sampling

The population consists of all professional nurses employed in the critical care units of the five selected public hospitals. Nurses in the study setting share a common foundational knowledge in managing central venous catheters, with particular emphasis on tunnelled central venous catheters – the most used type in this context. Their training covers all stages of catheter management – including preparation for insertion, assistance during insertion and post-insertion care. However, despite this foundational training, many nurses often lack ongoing in-service education to reinforce and update their skills. A convenience sample of 122 nurses was included in the study. The intervention group included nurses from three critical care units (1, 2 and 4) in three hospitals (comprising two tertiary hospitals and one regional hospital). The control group consisted of critical care units 3 and 5 in two hospitals (one tertiary hospital and one regional hospital). The intervention group units were in close geographic proximity (in one city), as were the control group units (in another city, 300 km from the city in which the intervention group units were located). The large geographical distance between the intervention and control groups assisted in limiting contamination of the study.

**FIGURE 1:**
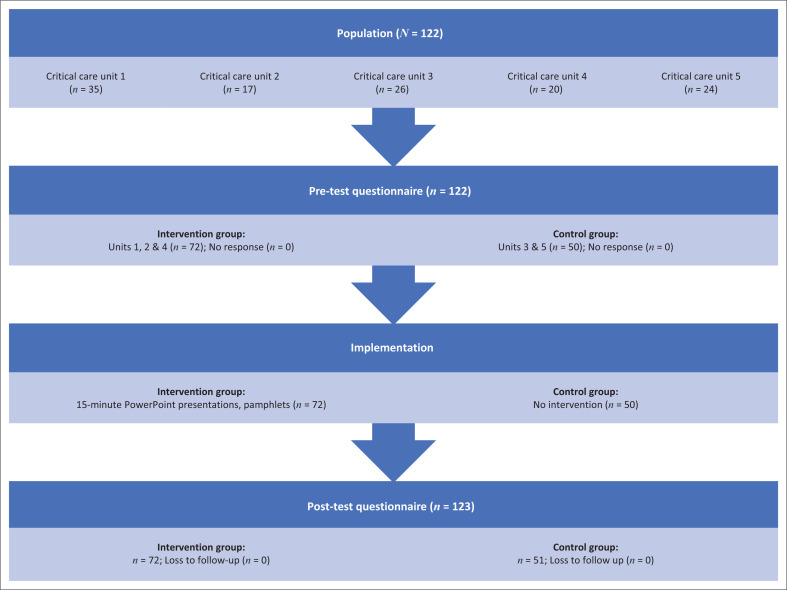
Sampling framework per group.

Figure 1 outlines the sample framework per group.

### Recruitment

After obtaining written permission from the hospital Chief Executive Officers and verbal consent from the unit managers, a date and time were scheduled, and a venue was assigned for the first author to present the study to the participants. Critical care nurses in both groups were informed of the study objectives and requirements to complete two questionnaires. Additionally, for the nurses in the intervention group, the intervention’s content and duration were communicated. Participation was voluntary and anonymous, and no incentives were provided.

### Educational intervention

The first author developed an educational intervention based on a clinical practice guideline for the management of central venous catheters in critically ill patients, as outlined by Zhang et al. (2018) for healthcare professionals in China, along with supporting literature. The intervention was tailored based on the results of the pre-test, with its design (layout, formulation) and content (feasibility and relevance) reviewed by three expert reviewers in critical care. The intervention aimed to provide critical care nurses with an overview of central venous catheter management before, during and after catheter insertion.

Table 1 outlines the details of the intervention.^1^

**TABLE 1:** Details of the educational intervention.

Educational strategies	Content (O’Grady et al. 2011; Septimus and Moody 2016; Zhang et al. 2018)
Nine-slide PowerPoint presentation, presented in a 20-min, face-to-face, interactive training session	Pictures and text, outlining the following regarding central venous catheters: ■ Definition ■ Indications ■ Common sites for insertion ■ Recommendation regarding site selection ■ Complications ■ Care bundles before, during and after insertion to prevent complications ■ Summary of points to remember on catheter care
Four-page, hard-copy brochure, distributed to participants immediately after the presentation as part of the training session	Pictures and text summarising the following aspects of central venous catheters: ■ Pictures and text, outlining the following regarding central venous catheters: ■ Definition ■ Indications ■ Common sites for insertion ■ Complications ■ Care bundles ■ Recommendations and principles of catheter care before, during and after insertion ■ Summary of points to remember on catheter care
Monitoring visits using face-to-face, 15-min group sessions, once a month per unit, for two months	Providing educational support to participants by asking about their understanding of the implementation process and the content shared through the training session and brochure, in order to clarify any misunderstandings about the content.

Please see full reference list of this article: Notshe, T., Ten Ham-Baloyi, W. & James, S., 2025, ‘Improving critical care nurses’ knowledge of central venous catheter management through education’, *Health SA Gesondheid* 30(0), a2966. https://doi.org/10.4102/hsag.v30i0.2966

In this study, a combination of multifaceted educational strategies – comprising both active (monitoring visits) and passive (training sessions and brochure distribution) approaches – was employed, as such strategies have been shown to be effective and feasible for implementing training aimed at improving adherence to best practices in high-demand healthcare environments such as critical care units (Thapa, Liu & Chair 2023; Trogrlic et al. 2020).

The educational intervention was implemented by the first author on a critical care nurse with over 20 years of experience, who was not employed at any of the hospitals in the intervention group (Booysen et al. 2024). The intervention lasted for 8 weeks, from 01 September 2020 to 31 October 2020.

### Pre- and post-questionnaires

As no suitable existing questionnaire was identified, a structured pre-test–post-test questionnaire was developed by the first author in consultation with an experienced statistician. This self-developed instrument was based on the clinical practice guideline by Zhang et al. (2018) and further supported by a comprehensive review of the literature on central venous catheter management (Notshe 2022). The questionnaires comprised two sections: the demographic profile of the participants (Section A), which comprised five questions related to respondents’ gender, age, number of years working in the critical care unit, critical care qualifications and educational programmes attended, such as in-service training sessions. Section B comprised 51 questions with items measuring nurses’ knowledge of central venous catheter management before (34 items), during (9 items) and after insertion (8 items), where the participants were to select ‘Yes’ (confirming the statement was true), ‘No’ (confirming the statement was false) or ‘Don’t know’ (when participants were unsure whether a statement was true or false). Knowledge scores were calculated as the percentage of correct responses for the set of items related to before, during and after insertion.

### Data collection

After obtaining written consent, participants were given a hard copy of the pre-test questionnaire, which was collected immediately upon completion. The educational intervention training session then commenced as part of the intervention process. To ensure that care was not compromised, two sessions were held in each hospital on the same day for the purposes of recruitment, data collection and conducting the educational intervention. Participants were instructed to refrain from communicating with one another during the data collection process to ensure the integrity of the data. The pre-test was conducted from 22 July 2020 to 31 August 2020, considering the geographical dispersion of the hospitals as well as additional restrictions and precautions related to the coronavirus disease 2019 (COVID-19) pandemic in South Africa. The post-test employed a data collection approach similar to that of the pre-test and was administered to both the intervention and control groups immediately following the completion of the 8-week implementation period for the intervention group. Data collection for the post-test took place between 01 November 2020 and 10 December 2020.

### Data analysis

The first author manually entered data from hard copy questionnaires into Excel and then cleaned it for accuracy and consistency. This process involved excluding incomplete or duplicate entries and correcting inaccurately entered responses, ensuring a reliable dataset for analysis. Subsequently, a statistician was involved to verify the data and to perform various statistical analyses using SPSS 13.3, including descriptive analysis, calculating measures of central tendency and dispersion, creating frequency distributions and conducting inferential statistics such as one-sample *t*-tests and analyses of variance (ANOVAs). *T*-tests were run to determine whether the improvement in knowledge (post- minus pre-scores) was statistically significant, and ANOVAs were used to determine whether one or more of the demographic variables were related to improvement in knowledge (Booysen et al. 2024). A *p*-value of less than 0.05 was considered to be statistically significant.

Cohen’s d was used to determine the practical significance of statistically significant results. For Cohen’s d, the following interpretation intervals were used: *d* < 0.20 (not significant); 0.20 ≤ *d* < 0.50 (small); 0.50 ≤ *d* < 0.80 (medium) and *d* ≥ 0.80 (large) (Gravetter et al. 2021).^2^

### Reliability and validity

To establish validity, the pre-test–post-test questionnaire was reviewed prior to data collection by three experts – two experienced intensivists and a critical care nurse with extensive clinical expertise. Additionally, the questionnaires were reviewed by a statistician. To further assess clarity and feasibility, the questionnaires were pilot tested with *n* = 14 nurses from a critical care unit at a smaller district hospital not included in the main study. Following the review and testing, it was necessary to provide clearer and more emphasised instructions for completing all the items. No changes to the content of the questions needed to be made. Cronbach’s alpha scores ranging from acceptable to strong were obtained across the various knowledge sections: 0.88 before insertion, 0.70 during insertion, 0.67 after insertion and 0.84 for all knowledge items.

### Ethical considerations

Ethical clearance was obtained from the university’s Faculty of Postgraduate Studies Committee (ethical clearance reference number H19-HEA-NUR-003) as well as from the Provincial Department of Health (reference number: EC_201904_013). Ethical principles such as informed consent and maintenance of confidentiality and anonymity were adhered to throughout the study. The data collected were stored by the second author in a locked cabinet and on a password-protected computer as per institutional policy. Upon completion of the study, the educational intervention was shared with the control group.

## Results

### Demographic data

All (100%) of the available critical care nurses (*n* = 122) in the five critical care units participated in both the pre- and post-tests. The control group had one additional critical care nurse who partook in the post-test because of a vacant post that was filled in the critical care unit 5 during the time the post-test was conducted. As the questionnaire was anonymous, it was not possible to identify and remove the additional participant from the data. Most of the participants were female nurses (*n* = 113, 92%), almost half of the participants fell in the 50–64 year age category (*n* = 58, 47%), and about two-thirds of participants had between 10 and more than 20 years of experience (*n* = 78, 64%). Slightly over half of the participants indicated that they had a qualification in critical care (*n* = 63, 51%). Most participants indicated not to have attended any educational programmes on the management of central venous catheters in the last 6 months (*n* = 100, 81%). No significant differences between the demographics in the control and intervention groups were noted (see Table 2).

**TABLE 2:** Demographic profiles of the intervention and control group participants.

Demographic items	Control		Intervention		Total	
	*n*	%	*n*	%	*n*	%
**Gender**						
Female	45	88	68	94	113	92
Male	2	4	4	6	6	5
No response	4	8	0	0	4	3
Total	51	100	72	100	123	100
**Age category (years)**						
23–39	13	26	16	22	29	24
40–49	15	29	21	29	36	29
50–64	23	45	35	49	58	47
Total	51	100	72	100	123	100
**Years working in a critical care unit**						
< 5	24	39	10	46	34	27
5–9	3	11	8	9	11	9
10–19	12	25	27	21	39	32
20 and more	12	25	27	24	39	32
Total	51	100	72	100	123	100
**Qualification in critical care**						
Yes	28	55	35	49	63	51
No	23	45	37	51	60	49
Total	51	100	72	100	123	100
**Attended educational programmes on the management of central venous catheters in the last 6 months**						
Yes	11	22	12	17	23	19
No	40	78	60	83	100	81
Total	51	100	72	100	123	100

### Knowledge

The participants’ knowledge related to managing central venous catheters before, during and after insertion is summarised in Table 3. The *t*-test results, presented in Table 3 and Table 4, were conducted to assess the significance of the mean knowledge score differences between the pre- and post-tests for both the control and intervention groups.

**TABLE 3:** Mean knowledge score differences between pre and post groups by control and intervention groups.

Score	Group	*n*	Mean	s.d.	Post – Pre Difference	*t*	*p* (*df* = 59)	Cohen’s *d*
**Control group**								
Before insertion	Pre	50	59.93	12.38	23.83	8.61	**< 0.0005**	**2.21**
	Post	51	83.76	9.20	-	-	**-**	**Large**
During insertion	Pre	50	47.68	12.62	23.84	5.59	**< 0.0005**	**1.44**
	Post	51	71.52	19.32	-	-	**-**	**Large**
After insertion	Pre	50	60.39	21.05	33.30	7.66	**< 0.0005**	**1.97**
	Post	51	93.70	12.42	-	-	**-**	**Large**
Knowledge overall	Pre	50	55.89	11.45	27.02	9.63	**< 0.0005**	**2.47**
	Post	51	82.91	10.45	-	-	**-**	**Large**
**Intervention group**								
Before insertion	Pre	72	74.07	11.38	23.94	18.55	**< 0.0005**	**2.74**
	Post	72	98.01	4.61	-	-	**-**	**Large**
During insertion	Pre	72	62.74	15.26	33.70	16.68	**< 0.0005**	**2.46**
	Post	72	96.44	11.85	-	-	**-**	**Large**
After insertion	Pre	72	75.50	15.41	21.54	12.43	**< 0.0005**	**1.83**
	Post	72	97.04	5.86	-	-	**-**	**Large**
Knowledge overall	Pre	72	70.80	10.04	26.34	22.15	**< 0.0005**	**3.27**
	Post	72	97.13	5.25	-	-	-	**Large**

Note: Bold values indicate significance of a *p*-value (< 0.0005) or Cohen’s *d*.

*df*, degrees of freedom.

**TABLE 4:** Mean pre-test knowledge score differences between the control and intervention groups.

Score	Group	*n*	Mean	s.d.	Int. – Con. difference	*t*	*p* (*df* = 59)	Cohen’s *d*
Before insertion	Con.	50	59.93	12.38	14.15	5.66	**< 0.0005**	**1.21**
	Int.	72	74.07	11.38	-	-	**-**	**Large**
During insertion	Con.	50	47.68	12.62	15.07	4.76	**< 0.0005**	**1.37**
	Int.	72	67.74	15.26	-	-	**-**	**Large**
After insertion	Con.	50	60.39	21.05	15.11	4.17	**< 0.0005**	**0.91**
	Int.	72	75.50	15.41	-	-	**-**	**Large**
Knowledge overall	Con.	50	55.89	11.45	14.91	6.67	**< 0.0005**	**1.44**
	Int.	72	70.80	10.04	-	-	**-**	**Large**

Note: Bold values indicate significance of a *p*-value (< 0.0005) or Cohen’s *d*.

Con., control; Int., intervention; *df*, degrees of freedom.

According to Table 3, both groups showed significant improvements (*p* < 0.0005) in knowledge. While the range of mean pre-post differences for the control group (23.83–33.30) closely mirrors that of the intervention group (21.54–33.70), it is important to note that the control group’s mean pre-test knowledge scores (47.68–60.39) were significantly lower than those of the intervention group (62.74–75.50), as shown in Table 4.

Table 5 outlines a comparison of the mean score differences across the questionnaire domains between the intervention and control groups.

**TABLE 5:** Mean score differences per questionnaire domain between the intervention and control groups.

Domain	Control group mean difference	Post – Pre	Intervention group mean difference	Post – Pre	Comparison	Significance*
Before insertion	+23.83	83.76–59.93	+23.94	98.01–74.07	Very similar mean increase	Large effect sizes
During insertion	+23.84	71.52–47.68	+33.70	96.44–62.74	Intervention group improved more	Large effect sizes
After insertion	+33.31	93.70–60.39	+21.54	97.04–75.50	Control group improved more here	Large effect sizes
Knowledge overall	+27.02	82.91–55.89	+26.33	97.13–70.80	Very similar overall improvement	Large effect sizes

* , *p*-value < 0.0005.

According to Table 5, both groups showed significant, large knowledge gains across all domains (*p* < 0.0005), with the intervention group improving more in the ‘during insertion’ domain, while the control group improved more in the ‘after insertion’ domain; overall improvements were similarly strong in both groups.

To determine whether the improvement in knowledge for the intervention group was greater than that of the control group while accounting for the lower pre-scores of the control group and potential demographic variables, ANOVAs were conducted. The results are presented in Table 6.

**TABLE 6:** Analyses of variance results – Knowledge scores before, during and after insertion between groups and demographic variables.

Score per domain or demographic variables	*F*-value	*df*	*p*-value	Cohen’s *d*
**Before insertion**				
**Between groups**				
Between intervention and control groups	88.54	1; 225	< 0.0005	0.83
Between pre- and post-groups	405.89	1; 225	< 0.0005	2.10
**Demographic variables**				
Age category	0.90	2; 225	0.407	n/a
Years working in a critical care unit	1.10	3; 225	0.351	n/a
Qualification in critical care	2.62	1; 225	0.107	n/a
Attended educational programmes on the care of CVCs in the last 6 months	0.03	1; 225	0.858	n/a
**During insertion**				
**Between groups**				
Between intervention and control groups	63.52	1; 225	< 0.0005	0.84
Between pre- and post-groups	256.06	1; 225	< 0.0005	1.80
**Demographic variables**				
Age category	0.50	2; 225	0.606	n/a
Years working in a critical care unit	0.06	3; 225	0.979	n/a
Qualification in critical care	0.05	1; 225	0.820	n/a
Attended educational programmes on the care of CVCs in the last 6 months	0.90	1; 225	0.344	n/a
**After insertion**				
**Between groups**				
Between intervention and control groups	14.34	1; 225	< 0.0005	0.38
Between pre- and post-groups	204.71	1; 225	< 0.0005	1.74
**Knowledge scores versus demographic variables**				
Age category	1.07	2; 225	0.344	n/a
Years working in a critical care unit	1.13	3; 225	0.338	n/a
Qualification in critical care	3.31	1; 225	0.070	n/a
Attended educational programmes on the care of CVCs in the last 6 months	0.02	1; 225	0.886	n/a

CVCs, central venous catheters; *df*, degrees of freedom.

As outlined in Table 6, based on the ANOVA results reported, none of the demographic variables had a significant effect on the observed improvement in knowledge.

## Discussion

This project aimed to describe the effect of an 8-week educational intervention on critical care nurses’ knowledge of the management of central venous catheters. The study results indicate that both groups showed significant improvements in knowledge. This finding is in contrast with similar studies using educational interventions to measure knowledge among nurses in critical care units, which often found an increase in knowledge only in the intervention groups (Almarhabi, Cornish & Lee 2021). The significant improvement in knowledge across both groups may reflect greater awareness of knowledge gaps among control group nurses, which in turn may have prompted self-directed learning through additional resources. This aligns with findings by Vasli and Asadiparvar-Masouleh (2024), who reported that self-directed learning can positively influence clinical competence.

Although overall knowledge significantly improved in both groups, the intervention group showed greater gains in the ‘during catheter insertion’ domain, while the control group improved more in the ‘after insertion’ domain. This improvement may be linked to previous exposure or training in specific areas of central venous management, highlighting the role of structured education in enhancing skills at critical moments. In addition, despite no significant differences between the groups in terms of prior education and training on central venous catheters within the last 6 months, the intervention group demonstrated significantly higher pre-test knowledge compared to the control group. Several factors could explain this finding. Firstly, it is plausible that the intervention group exhibited superior retention of information owing to the quality of their education and/or training, their individual learning aptitudes or the frequency of their practice. Secondly, the intervention group may have benefited from supplementary learning opportunities, such as access to additional reading materials, participation in online courses or engagement in hands-on practice sessions, which could have bolstered their understanding of central venous catheter procedures. Thirdly, the intervention group might have demonstrated greater motivation and engagement during their prior education and training sessions, leading to more effective absorption and internalisation of the material presented. Several studies in critical care settings support the finding that knowledge levels can be influenced despite similar prior training. Aloush (2018) and İskender and Karadeniz (2025) found that the quality and structure of education significantly impact knowledge retention. Additionally, access to supplementary learning methods, such as e-learning, has been shown to enhance understanding of central venous catheter care (Foka et al. 2023), which could explain the intervention group’s higher pre-test scores. However, within the context of this study, further exploration through qualitative methods or follow-up assessments could offer valuable insights into the underlying factors contributing to the observed differences in pre-test knowledge between the two groups.

Furthermore, none of the demographic factors were found to be significantly related to the improvement in knowledge between the pre- and post-tests. This finding contrasts with a cross-sectional study by Indarwati, Munday and Keogh (2022), which identified a positive correlation between educational qualifications, training and work experience – all of which were significantly associated with critical care nurses’ knowledge and confidence regarding catheter use.

Although it is evident from the findings that the 8-week educational intervention has the potential to significantly improve critical care nurses’ knowledge of central venous catheter management, further adaptation and evaluation of the intervention may be beneficial. Incorporating additional teaching strategies such as simulation-based learning, video demonstrations and checklists could strengthen its effectiveness. For instance, simulation-based education has been shown to significantly improve technical performance in central venous catheter management (Ma et al. 2011), while video-based training has been found to enhance adherence to sterile techniques during catheter insertion (Xiao et al. 2007). Moreover, integrating a catheter management checklist into the intervention could support knowledge retention and help reduce the incidence of procedure-related complications, including catheter-related infections (Aloush 2018). Formal education, along with ongoing training through workshops, on the management of central venous catheters should also be provided to nurse educators. This is particularly important given that half of the study participants reported not holding a qualification in critical care, and the majority had not received any prior training in catheter management.

Ongoing education, orientation and mentoring on the management of central venous catheters – grounded in evidence-based guidelines – are essential, as recommended by a similar intervention study (Sharour et al. 2018). Such continuous professional development can not only improve nurses’ knowledge but also strengthen their confidence in adhering to best practices for catheter management. However, successful central venous catheter management requires institutional support in the form of dedicated time, resources and strong leadership, for example, through initiatives like champion teams (Reed, Brock & Anderson 2014) or nurse-led interdisciplinary strategies like the Vascular Access Specialist Team, comprising senior nurses, technologists and physicians (Yin et al. 2023).

Overall, the study findings have significance for nurse leaders, especially within the critical care context. Further, the study findings can be used to shape the training curriculum and continuous professional development programmes in critical care nursing. Furthermore, the findings can contribute to the empowerment of clinical staff by enhancing their knowledge of central venous catheter management. This increased competence may lead to improved clinical practices, a reduction in catheter-related complications and, ultimately, better outcomes for critically ill patients. By providing opportunities for critical care nursing staff to enhance their knowledge, an organisation demonstrates a clear commitment to prioritising the quality of patient care.

### Strengths and limitations of the study

This study has several limitations. Initally, the study lacks random sampling methods and sample size calculation, which affects its generalisability. Additionally, the sample size is relatively small. The use of self-administered questionnaires may introduce response bias. Follow-up testing was not conducted, and the impact of the intervention on patient outcomes – such as length of hospital stay and the incidence of complications – was not assessed, representing a limitation of the study. Furthermore, during recruitment and data collection, additional measures had to be implemented to adhere to COVID-19 infection control principles, potentially influencing our findings.

### Recommendations for further research

To address these limitations, future research should include a larger sample size and assess the long-term impact of the intervention on both nurses’ knowledge and patient outcomes. This approach would facilitate the further development and refinement of the educational intervention, providing a more comprehensive evaluation of its effectiveness in improving critical care nurses’ knowledge and clinical practices in central venous catheter management. In addition, it is recommended that future research explore the impact of educational interventions on key central venous catheter-associated complications within critical care settings, tailored to the specific contexts in which they are implemented.

## Conclusion

The findings of the research study demonstrated that an 8-week educational intervention has the potential to improve critical care nurses’ knowledge of central venous catheter management. Knowledge improved in both the intervention and control groups, with the intervention group improving more in the ‘during catheter insertion’ domain and the control group more in the ‘after catheter insertion’ domain. Notably, the intervention group demonstrated significantly higher pre-test knowledge compared to the control group. Further exploration is needed to identify the factors that facilitate improved knowledge among critical care nurse participants, as well as to adapt to and rigorously test the intervention. This should include the incorporation of diverse educational strategies, such as simulation-based learning, video demonstrations and catheter care checklists, to enhance the effectiveness and applicability of the training. In addition, orientation, mentoring and formal training are recommended, which require institutional support and leadership.

## Footnotes

1 Kindly note that the control group did not receive any intervention.

2 Note that inferential statistics for the pre-test knowledge data were conducted for the *n* = 72 sample who also participated in the post-test.
